# A Wonderful House

**Published:** 1874-12

**Authors:** 


### A WONDERFUL HOUSE.

We allude to the house of R. H. Macy & Co., on the corner of Fourteenth Street and Sixth Avenue. To us it is a wonderful curiosity. It is more than this; it is an ever-increasing wonder. Here are acres of space occupied by myriads of useful and ornamental articles, at prices marvellously low. Five hundred and twenty-eight attendants wait upon the multitude of customers who hourly throng the great stores and jostle each other in their pursuit of the bargains offered in the sixteen departments into which the immense stock is divided.

There is probably no store in America at all equal to this in the variety of goods displayed. The sixteen departments alluded to comprise goods which a closely-printed catalogue of 115 pages suffices to enumerate but imperfectly. The store is a perfect museum in itself. There are white goods and house furnishing articles in amazing variety; laces, embroideries, cuffs, collars and ruffles, corsets, handkerchiefs and underwear—an endless catalogue; underclothing of all kinds, and children's dresses; notions and smallwares, which our entire magazine would not afford space to enumerate; millinery goods of every description; silver-plated ware from the best manufactories; gloves, hosiery, underwear, gents' and boys' furnishing, and umbrellas; ladies' ties, parasols, sun umbrellas; toilet and traveling furnishing goods, leather goods, medicines and druggists' sundries; jewelry and fancy goods, bronzes and parians; every variety of house furnishing goods; toys in vast quantity, including dolls, whose perfection approaches that of nature, and which vary in size from one inch to five feet long; books, stationery, velvet frames, stereoscopes, views, albums—this department forming a more complete book-store than many establishments devoted exclusively to the business; worsted and worsted goods, trimmings, fringes and buttons; china, glass, earthen, Rockingham, majolica, Java, parian and Limoge ware, forming a new and most interesting department, which embraces a world of wonderful art; products in china and other imported wares, together with beautiful glass ware in abundant variety. Following all these wonders we have a pic-nic and confectionery department, the former supplied with all kinds of prepared goods, pickles, sauces, pateoxolims, canned and preserved goods, teas, coffees, and biscuits; the latter embracing all the choice candies and confections known in Europe or America.

We must not omit to allude to an important specialty of the house—the La F orge Glove. This is claimed by many wearers to be the cheapest, strongest, and most durable kid glove in use. It is certainly a very soft and beautiful glove, and is fully warranted by the house.

Our list, of course, is very incomplete, but enough has been said to show such of our readers as are unfamiliar with the interior of this vast establishment that nearly all the wants of the shopper can here be supplied; to the residents of New York and vicinity not a word is necessary. On every fine afternoon the streets in the region of this store are crowded with the fine carriages of purchasers. Multitudes visit the place as they would any other great museum of wonders, to feast the eye upon the charming display; and, though the motive may be simply that of curiosity, few, if any, leave without bearing away some of the numerous useful and attractive things displayed. It is difficult to resist the impulse to buy, since no housekeeper, however amply supplied with articles of use and adornment, can pass through the crowded halls without discovering something tempting, unique, and desirable, which the proprietors have seemingly ransacked the world to find. There is no invention of use in the kitchen, no labor-saving appliance connected with housekeeping, which does not find a place here; and, after we had visited the recent Exhibition of the American Institute, we remarked that if that excellent institution could prevail on Mr. Macy to contribute thereto a hundredth part of his novelties, its interest would be enhanced a hundred-fold.

As may be inferred, the business done by this house is enormous. It has won its way by great enterprise in the selection of choice goods, by low prices, and by the most unexceptional fair dealing. We have traded at this house for years, and hardly a day passes in which something is not bought at Macy's for household use. We hardly know the proprietor—the organizer of this vast concern—by sight, but we have long since discovered that it is profitable to patronize his mammoth house ; and we do not hesitate to send abroad this testimony on behalf of one who abundantly deserves such encomiums as it is in our power to bestow.

Long may the "Red Star" of Macy remain in the ascendant to direct the purchaser to the spot where nearly all things good and desirable may be found under one roof.

Our readers residing at a distance may be glad to know that the great descriptive catalogue will be sent, postpaid, on application, and that the house does a very extensive business through the mails. Packages are sent by mail at the rate of two cents for four ounces, and one cent for each additional two ounces, or fraction thereof, under four pounds.

---

By subjecting seaweed to distillation with superheated steam, according to Stanford's process, instead of simply reducing it to ashes, as has hitherto been done, not only can illuminating gas, acetic acid, and combustible oils be obtained, but iodine, chloride of potassium, etc., can be extracted from the residue. The charcoal residuum also possesses an unusual deodorizing power, and can be used for disinfecting water closets in such manner as to constitute a source of ammonia, by further distillation.

